# Automated Monitoring of Adherence to Evidenced-Based Clinical Guideline Recommendations: Design and Implementation Study

**DOI:** 10.2196/41177

**Published:** 2023-05-04

**Authors:** Gregor Lichtner, Claudia Spies, Carlo Jurth, Thomas Bienert, Anika Mueller, Oliver Kumpf, Vanessa Piechotta, Nicole Skoetz, Monika Nothacker, Martin Boeker, Joerg J Meerpohl, Falk von Dincklage

**Affiliations:** 1 Department of Anesthesia, Critical Care, Emergency and Pain Medicine Universitätsmedizin Greifswald Greifswald Germany; 2 Department of Anesthesiology and Operative Intensive Care Medicine Charité – Universitätsmedizin Berlin, corporate member of Freie Universität Berlin and Humboldt-Universität zu Berlin Berlin Germany; 3 Institute of Medical Informatics Charité – Universitätsmedizin Berlin, corporate member of Freie Universität Berlin and Humboldt-Universität zu Berlin Berlin Germany; 4 Einstein Center Digital Future Berlin Germany; 5 Evidence-based Medicine, Department I of Internal Medicine Faculty of Medicine and University Hospital Cologne University of Cologne Cologne Germany; 6 Institute for Medical Knowledge Management Association of the Scientific Medical Societies University of Marburg Marburg Germany; 7 Institute for Artificial Intelligence and Informatics in Medicine Medical Center rechts der Isar, School of Medicine Technical University of Munich Munich Germany; 8 Institute for Evidence in Medicine Medical Center & Faculty of Medicine University of Freiburg Freiburg Germany; 9 Cochrane Germany Cochrane Germany Foundation Freiburg Germany

**Keywords:** clinical decision support, evidence-based medicine, computer-interpretable guidelines, COVID-19, clinical guideline recommendations, monitoring, clinical, patient, prototype, utility, data, system

## Abstract

**Background:**

Clinical practice guidelines are systematically developed statements intended to optimize patient care. However, a gapless implementation of guideline recommendations requires health care personnel not only to be aware of the recommendations and to support their content but also to recognize every situation in which they are applicable. To not miss situations in which recommendations should be applied, computerized clinical decision support can be provided through a system that allows an automated monitoring of adherence to clinical guideline recommendations in individual patients.

**Objective:**

This study aims to collect and analyze the requirements for a system that allows the monitoring of adherence to evidence-based clinical guideline recommendations in individual patients and, based on these requirements, to design and implement a software prototype that integrates guideline recommendations with individual patient data, and to demonstrate the prototype’s utility in treatment recommendations.

**Methods:**

We performed a work process analysis with experienced intensive care clinicians to develop a conceptual model of how to support guideline adherence monitoring in clinical routine and identified which steps in the model could be supported electronically. We then identified the core requirements of a software system to support recommendation adherence monitoring in a consensus-based requirements analysis within the loosely structured focus group work of key stakeholders (clinicians, guideline developers, health data engineers, and software developers). On the basis of these requirements, we designed and implemented a modular system architecture. To demonstrate its utility, we applied the prototype to monitor adherence to a COVID-19 treatment recommendation using clinical data from a large European university hospital.

**Results:**

We designed a system that integrates guideline recommendations with real-time clinical data to evaluate individual guideline recommendation adherence and developed a functional prototype. The needs analysis with clinical staff resulted in a flowchart describing the work process of how adherence to recommendations should be monitored. Four core requirements were identified: the ability to decide whether a recommendation is applicable and implemented for a specific patient, the ability to integrate clinical data from different data formats and data structures, the ability to display raw patient data, and the use of a Fast Healthcare Interoperability Resources–based format for the representation of clinical practice guidelines to provide an interoperable, standards-based guideline recommendation exchange format.

**Conclusions:**

Our system has advantages in terms of individual patient treatment and quality management in hospitals. However, further studies are needed to measure its impact on patient outcomes and evaluate its resource effectiveness in different clinical settings. We specified a modular software architecture that allows experts from different fields to work independently and focus on their area of expertise. We have released the source code of our system under an open-source license and invite for collaborative further development of the system.

## Introduction

### Background

Clinical practice guideline recommendations are intended to optimize patient care by assisting the decision-making of health care professionals within specific clinical circumstances [1-3]. Thus, clinical practice guideline recommendations are among the most important potential clinical decision support (CDS) tools [4,5]. Considering and implementing such recommendations during patient management is expected to be associated with improved patient outcomes, especially in the case of evidence-based recommendations that were developed based on systematic reviews and appraisal of the available evidence [6-8]. However, a gapless implementation of clinical practice guideline recommendations in daily routine work requires health care professionals not only to be aware of the existence of the respective guideline recommendations, to understand and support their content, but also to correctly recognize all situations in which specific recommendations should be applied [9].

Meeting the latter requirement becomes particularly demanding in the interdisciplinary treatment of patients with complex conditions that affect multiple organ systems, as it is often the case in critical care medicine [10-12]. Ensuring that all health care professionals have active knowledge about all guideline recommendations that apply in such situations and that they correctly recognize every situation in which these recommendations should be applied can prove difficult. Thus, treatment in critical care medicine is at a comparably high risk of deviating from guideline recommendations [10].

Besides the multitude of simultaneously applicable guideline recommendations in critical care, another aspect that can strongly affect guideline recommendation adherence is the high frequency of changes in recommendations [11]. The COVID-19 pandemic presented an exemplary situation in which the dissemination and implementation of guidelines via conventional processes struggled to keep pace with the rapid development of recommendations and the speed at which recommendations were updated and changed over time [13,14].

To counter such difficulties and assist in the implementation of clinical guideline recommendations using computerized CDS, various machine-readable guideline recommendation formalisms have been developed [15-22]. However, these formalisms focus on representing finalized guideline recommendations and do not consider the systematic development process from which evidence-based guideline recommendations are derived. We have recently developed a *Fast Healthcare Interoperability Resources* (FHIR)–based formalism for the computer-interpretable representation of the whole guideline recommendation development process by developing systematic reviews of primary studies, rating the certainty of the available body of evidence, and finally applying evidence to decision frameworks to derive the final recommendation, called *Clinical Practice Guidelines on Evidence-Based Medicine on FHIR* (CPG-on-EBMonFHIR; [23]). This formalism is geared toward separate evidence-based guideline recommendations, which are often simple rules linking a specific condition to a specific action, as they are usually based on studies that focus on specific interventions and limited options rather than complex clinical pathways because of the logical complexity that can be studied in randomized trials.

### Objectives

We aimed to (1) collect and analyze the requirements for providing CDS via automated monitoring of individual evidence-based guideline recommendation adherence, (2) design and implement a prototype that fulfills the requirements, and (3) test the prototype’s applicability on real patient data.

## Methods

### Overview

**Figure 1:**
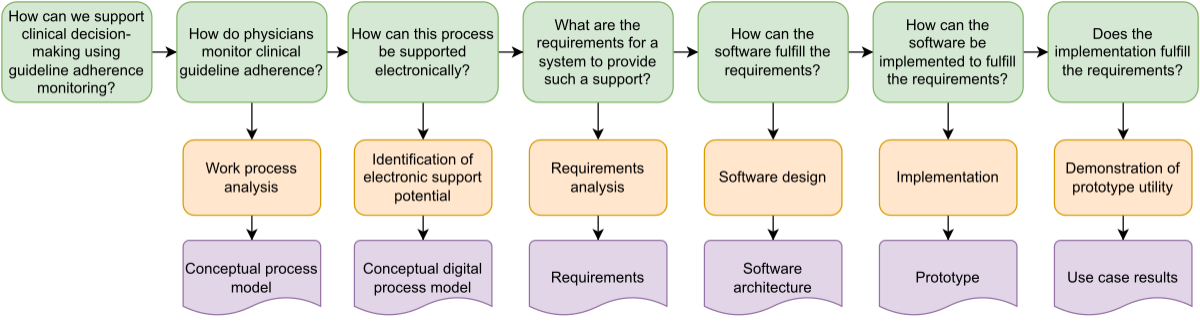
Flowchart of the study and the derived artifacts at each step.

To derive the requirements for a software system to monitor adherence to clinical guideline recommendations, we first performed a work process analysis of the clinical processes that are to be supported by the system (Figure 1). After identifying and structuring these processes, we determined which subprocesses can be supported electronically and identified the requirements for how these subprocesses can be supported by a software system. On the basis of these requirements, we designed a modular system architecture that we implemented as an open-source prototype. To demonstrate the utility of the prototype implementation, we applied it to monitor adherence to a COVID-19 treatment guideline recommendation based on clinical data from a large European university hospital.

### Work Process Analysis, Identification of Electronic Support Potential, and Requirements Analysis

To derive the requirements for the system, we first conducted a needs analysis for the users of the system, the clinical staff. This needs analysis was conducted as a work process analysis in which 5 experienced intensive care clinicians contributed to the flowchart modeling of how adherence to guideline recommendations would be monitored in clinical practice*.* The modeling was performed based on an iterative feedback process as follows:

1. Clinicians provided their input in personal or web-based meetings on how they would approach monitoring of guideline adherence in clinical practice.
2. The input was collected and consolidated by one of the participating clinicians, and a flowchart model was created to reflect the consensus of the group.
3. The model was then reviewed and discussed by clinicians in meetings or via email, and any necessary revisions were made based on the feedback provided.
4. The revised model was reviewed again by the clinicians, and the process continued until all clinicians agreed with the final outcome and had no further desired modifications.
5. The final model was used as the basis for the needs and requirements analysis, design, and development of the CDS system.

During the development of the conceptual model, there were some disagreements among the 5 clinicians involved in the process. These disagreements arose when discussing the steps and level of detail that should be included in the model based on their experience and individual clinical practice. However, these disagreements were reconciled through open and constructive dialog among the clinicians. The goal was to reach a consensus to ensure that the conceptual work process model accurately reflected general clinical practice.

On the basis of the work process model, we identified which parts of this process could be supported electronically and developed a flowchart model of how clinicians would interact with an electronic system to achieve the goal of monitoring patient-specific guideline recommendation applicability and adherence. The identification of support potential and process modeling was again performed in an iterative feedback process among the same group of clinicians, together with a health software architect.

To identify the requirements of the software system for monitoring individual recommendation applicability and adherence, we performed a comprehensive needs analysis for the system by involving key stakeholders:

1. Clinical staff, as they are the primary users of the system.
2. Clinical practice guideline developers, as they create and maintain the guideline recommendations that are used by the system.
3. Health data engineers familiar with hospital IT infrastructure, as the system is required to process data from electronic health records (EHRs).
4. Software developers, as they are required to build, test, and maintain the system.

Stakeholders were recruited by convenience from participants at the senior level of their respective field within the COVID-19 Evidence Ecosystem project of the Federal Network of University Medical Centers in Germany [24]. They were approached individually and requested to participate. No compensation was offered to participants. We required at least 2 participants per stakeholder group.

### Software Design and Prototype Implementation

The software prototype was implemented using an agile, rapid application development approach. The architecture followed a microservice pattern to allow efficient separation of concerns and scalable and exchangeable deployments of the system within heterogeneous clinical IT infrastructures. Each container exposes a RESTful application programming interface (API) specified according to the OpenAPI 2.0 standard [25]. Backend modules were implemented in Python 3.8 (Python Software Foundation) and the frontend modules using RShiny (RStudio) [26].

### Demonstration of Prototype Utility

To demonstrate the utility of the prototype, we connected it to anonymized clinical data from a large university hospital (Charité Universitätsmedizin Berlin, Germany) and integrated a recent strong evidence-based guideline recommendation for the treatment of patients with severe or critical COVID-19 [27].

### Ethics Approval

The use of anonymized clinical data for research was approved by the local ethics committee (Ethikausschuss 4 am Campus Benjamin Franklin, Charité Universitätsmedizin Berlin, Chairperson Prof R Stahlmann, Application Number EA4/008/19, approval date: February 6, 2019, amendment date: May 14, 2020).

## Results

### Work Process Analysis

**Figure 2:**
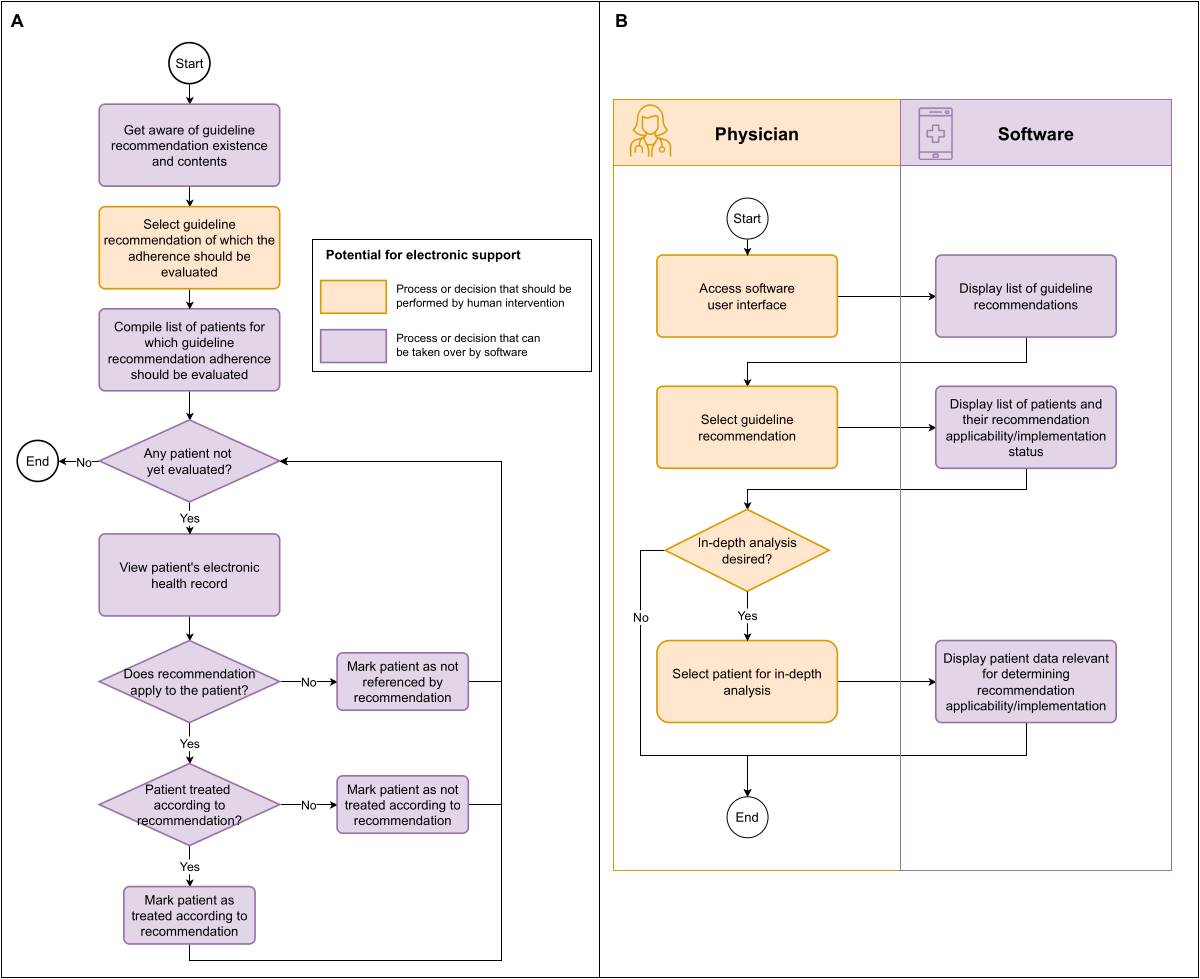
Conceptual process models of recommendation adherence monitoring in current clinical practice and using an electronic support system. (A) Conceptual work process model illustrating the steps involved in monitoring adherence to clinical guideline recommendations within the current clinical workflow. (B) Conceptual digital process model demonstrating the integration of an electronic decision support system to improve adherence monitoring in clinical practice.

To ensure that our system effectively supports the work processes of intensive care physicians, we conducted a comprehensive needs analysis with a group of key stakeholders. This included clinical staff made up of experienced intensive care physicians, as they are the primary users of the system, clinical practice guideline developers, health data engineers familiar with hospital IT infrastructure, and software developers. Together, they created a flowchart outlining the process of monitoring adherence to guideline recommendations (Figure 2A). The core insight of the work process analysis was that to evaluate and monitor guideline recommendation adherence, clinicians always work at the ward level and examine each patient individually to see whether a guideline recommendation applies and whether it is fulfilled.

### Identification of Electronic Support Potential

On the basis of the work process model, the clinical staff, together with a health software architect, identified which steps could be supported or covered by an electronic system. We identified nearly all steps as susceptible to being taken over by a software system (Figure 2A). The same group then determined how this process should be supported electronically and developed a corresponding model of the digitized work process (Figure 2B). The most important insight of this analysis was the necessity to display the raw patient data that underlies the system’s decision on recommendation applicability and adherence because clinicians using a software-based decision support system to monitor recommendation adherence want to be able to examine the raw patient data that underly the system’s decision on recommendation applicability and adherence to ensure that these data and decisions are correct, as EHR data may contain errors.

### Requirements Analysis

For a computer system that should support the defined work process (Figure 2A), we identified 4 core requirements in a series of focus work group feedback rounds.

#### Requirement 1: The System Needs to Be Able to Decide Whether a Guideline Recommendation Is Applicable and Whether a Guideline Recommendation Is Implemented for a Specific Patient

The task of checking whether a guideline’s recommendation is applicable and whether it is implemented for an individual patient or not requires the system to be able to process both the semantical (ie, what is the meaning of the words used in the recommendation) and logical (ie, which of the words used in the recommendation define who the recommendation applies to and which words define what is to be done or not to be done) content of the recommendation. Therefore, the system needs to be provided with guideline recommendations in a format that is semantically correct, complete, and unambiguous.

Here, we focus on evidence-based guideline recommendations, which makes the decoding of the logical content of the recommendations particularly easy: in the development process of evidence-based recommendations, it is standard practice to decompose the clinical question in consideration according to the PICO (population or patients, intervention, comparison, outcomes) framework [28]. Therefore, in these recommendations, the patients to which the guideline recommendation is applicable (P in PICO) and the intervention (I in PICO) that is recommended are distinctly defined at its best beginning with the systematic reviews supporting evidence-based guideline recommendations.

Regarding the decoding and interpretation of the semantical content of guideline recommendations, we developed a FHIR-based format for the representation of clinical practice guideline recommendations to provide an interoperable, standards-based guideline recommendation exchange format that fulfills the previously mentioned requirements [23]. Moreover, a variety of formalisms for representing guideline recommendations in a computer-interpretable manner exist [29-31], and any of these could be used, provided that they are able to represent guideline recommendations semantically correct, complete, and unambiguous.

#### Requirement 2: The System Needs to Be Able to Integrate Clinical Data From Different Data Formats and Data Structures

Despite a multitude of initiatives for standardization, patient data are often only available in proprietary and nonstandardized data formats and data structures that differ between countries, hospitals, or even wards in the same hospital. Therefore, to make the system applicable across various existing IT infrastructural settings, the second core requirement is that the data must be accepted in a standardized, interoperable format, into which all proprietary data formats can be converted. Among the data formats that fulfill these requirements are the Observational Medical Outcomes Partnership common data model [32] or FHIR-based formats (eg, the US Core Profiles [33] or the German Corona Consensus data set [34]).

#### Requirement 3: The System Needs to Automatically Adopt Changes in Clinical Guideline Recommendations

Clinical guideline recommendations are subject to change as medical knowledge advances. Considering the vast number of new findings being published in the medical literature every day and the subsequent frequency of guideline recommendation updates, any efforts to manually implement updated guideline recommendations in a software system can be expected to delay updates and pose a source of error [11].

One aspect that complicates error-free manual implementation of guideline recommendations in a software system is that such a task requires the expertise of at least 2 different and highly specialized fields: the expertise of the medical subspecialty providing the guideline recommendation and the software development expertise necessary for implementation into a system. Having both at one’s disposal for every single new or updated guideline recommendation is difficult and expensive.

Therefore, the system must adopt changes in the guideline recommendations without requiring changes in the software code of the system. Instead, once changes in guideline recommendations are released by responsible medical societies or other appropriate sources, these changes should be automatically adopted by the system without the requirement of any manual changes in the system’s software.

#### Requirement 4: The System Needs to Provide User Interfaces Optimized for Distinct User Groups

Different users of a CDS system require different user interfaces depending on the specific work processes that are to be supported by the system. For example, medical or nursing staff working on individual patients require a system that is highly integrated with the standard patient data visualization used during the treatment process (eg, the critical care information system used in the ward) to allow the seamless integration of decision support into individual patient care. In contrast, cross-section staff such as quality officers or supervising staff require more comprehensive overviews of multiple patients simultaneously, with less integration with other patient data, as their work processes that are to be supported by the system are more disconnected from individual patient care. Therefore, the fourth core requirement is that the user interface of the system must be customizable to meet the specific requirements that allow integration into the work processes of the respective groups of users that are to be supported.

### Prototype Implementation

#### Overview

**Figure 3:**
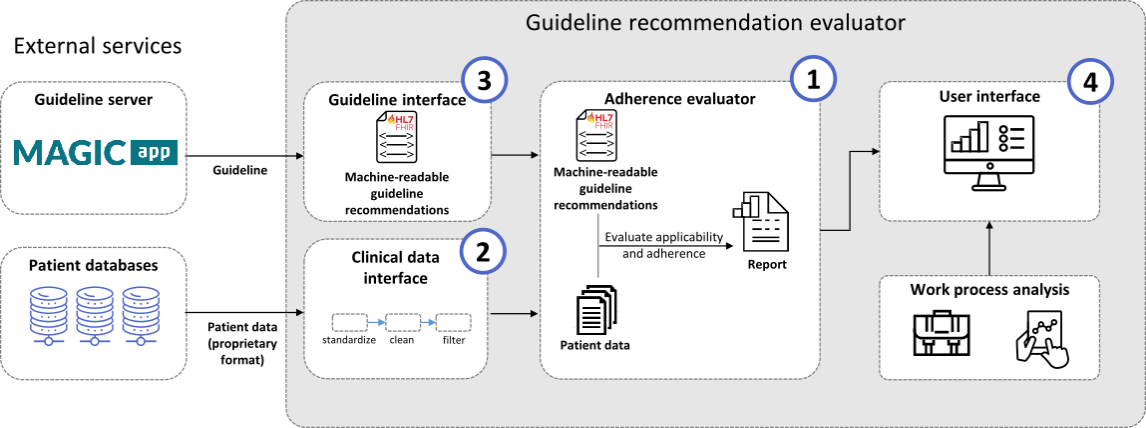
Architecture of the guideline recommendation evaluator for the automated integration of clinical guideline recommendations with real-time clinical data. Numbers indicate the requirement that is associated with the module.

Considering the advantages of a modular system in which each module corresponds to a specific specialty, we decided on a software architecture with 4 main modules that correspond to the previously described 4 main requirements, each of which requires the involvement of only 1 of the 3 stakeholder groups besides the software developers (Figure 3; Table 1). Each module is implemented as a separate microservice and exposes a REST API according to the OpenAPI 2.0 standard. To facilitate deployment and management, microservices are set up as Docker containers and can be orchestrated using Docker Compose. In the prototype implementation, the APIs are the fixed interfaces where the data requirements are set and expected. However, depending on the use case, they may need to be adapted to suit specific needs, for example, using a different data model for clinical data. Although these modules are independent of each other, they are designed to work in concert. However, in principle, they could be integrated with other services: the guideline interface could be used by any service requiring guideline recommendations in the computer-interpretable FHIR format. Likewise, the clinical data interface can be used by other services that require clinical data according to the implemented data model. The adherence evaluator can provide its results to different user interfaces, for example, depending on the clinical process that is to be supported.

**Table 1:** Overview of the prototype’s modules and their matching requirement.

Requirement	Module name	Description	Main stakeholder group
#1	Adherence evaluator	Reads and interprets guideline recommendations, requests required data from hospital database, and executes the conditions defined by the recommendations on the data	Software developers
#2	Data interface	Provides patient data in a standardized format and acts as converter or gateway to proprietary hospital databases	Health data engineers
#3	Guideline interface	Provides guideline recommendations in a computer-interpretable representation from a local or central repository	Clinical practice guideline developers
#4	User interface	Provides visualization and interactivity of the guideline recommendation evaluation results to end users and can be optimized for different end user groups	Clinical staff

#### Guideline Adherence Evaluation

The prototype implementation uses a core module to input guideline recommendations in a FHIR-based declarative format and builds executable event-condition-action rules based on the criteria specified in the recommendations. These rules are then executed against standardized patient data to determine whether the patients’ data align with the recommendations. The module, implemented in a Python package (Multimedia Appendix 1 [35-43]), selects 2 sets of criteria in the guideline recommendation, 1 for the patient population, which are addressed by the recommendation (eg, critically ill patients with COVID-19 requiring oxygen supply) and 1 for the recommended intervention for these patients (eg, daily administration of 1 mg dexamethasone for 10 days). The population and intervention parts are specified in a structured way in the relevant FHIR resources, and the individual semantic terms are coded using international standardized medical terminologies such as SNOMED CT, LOINC, ICD-10, ATC, or UCUM. These concepts must be mapped to the codes used in patient data to evaluate the applicability of the individual population and intervention criteria. Each individual criterion (eg, COVID-19; specific drug administration) is then evaluated and combined to yield patients that fulfill the population and intervention part of the recommendation. This allows for the evaluation of the applicability and adherence of guideline recommendations to individual patients. A more detailed description of the guideline adherence evaluator is provided in the Multimedia Appendix 1.

#### Clinical Data Interface

The role of the clinical data interface is to provide structured patient data from individual hospital EHR systems in a common data model for consumption by the adherence evaluator module. This interface needs to be implemented individually for each hospital EHR system to convert patient data from the local, mostly proprietary data models, into a common data model, thereby allowing the integration of guideline recommendations with patient data independent of the underlying local data model. In the prototype, we used a tabular format that incorporates standardized terminology for various clinical variables. However, the data could likewise be provided in an open standardized data model, such as the Observational Medical Outcomes Partnership common data model or openEHR-based or FHIR-based format. The relevant factor is that clinical data need to be provided in a common data model that is independent of the local data models of individual clinics’ EHR systems, which is understood by the adherence evaluator module. The minimal data requirements for evaluating adherence to a specific guideline recommendation depend on the specific medical concepts addressed within the recommendation. Adherence can be evaluated automatically only if all relevant clinical data can be retrieved from the EHR system in a structured format. In our prototype implementation, an extract, transform, and load connector was implemented for 3 different EHR systems to provide data that could be consumed by the adherence evaluator. Our prototype implementation uses a predefined list of patient data and must be adapted to the characteristics of individual hospital systems for use with real-time clinical data. A more detailed description of the clinical data interface is provided in the Multimedia Appendix 1.

#### Guideline Interface

The role of the guideline interface is to provide guideline recommendations in a machine-readable format to the adherence evaluator. As recommendations are represented in a declarative, computer-interpretable format using a structured representation of the population and intervention parts of the recommendation, with all criteria coded using international standardized terminologies, the adherence evaluator can use the same software code to evaluate adherence to the guidelines, regardless of the contents of the recommendations, provided that the data used in the recommendations are available in the hospital’s EHR systems. As the representation of the recommendations is consistent and uses standardized terminologies, the adherence evaluator can input the recommendations, generate computer-executable event-condition-action rules based on the recommendations contents, and evaluate these on patient data without the need for additional explicit software code. The guideline interface communicates in a unidirectional fashion with the adherence evaluator, sending only the guideline contents to the adherence evaluator. Updates to the guideline contents are provided to the guideline interface by the appropriate organization (eg, medical societies) that publishes the guidelines; therefore, no communication from the adherence evaluator to the guideline interface is necessary.

#### User Interface

**Figure 4:**
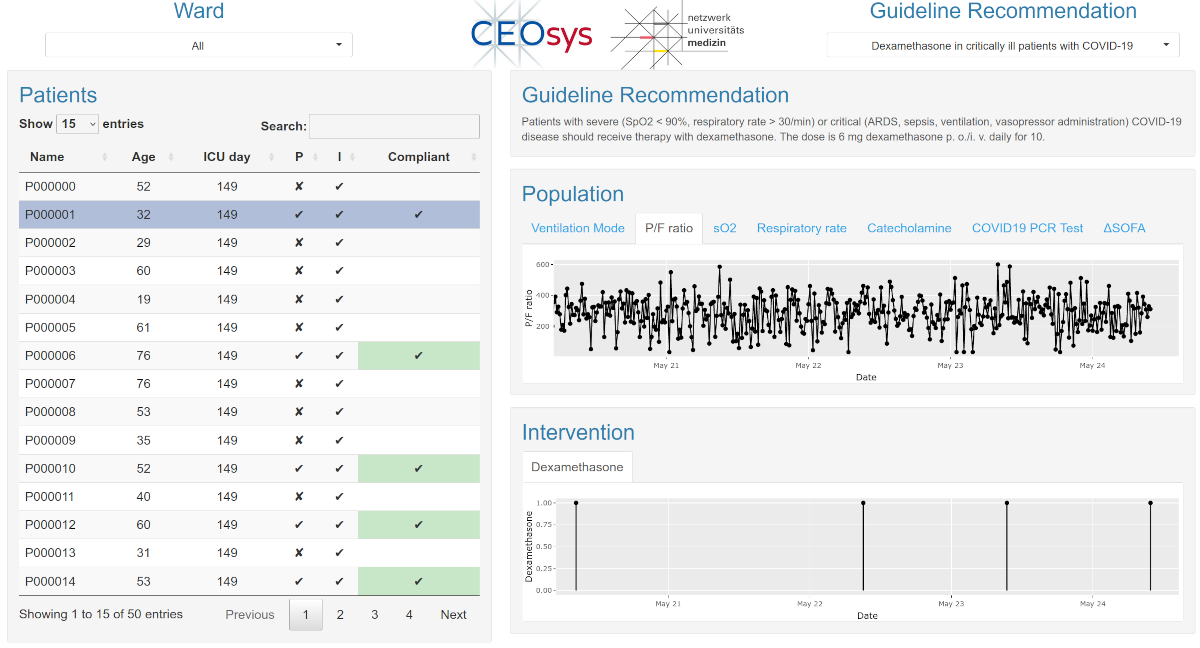
Prototype implementation of the user interface. The user can select a guideline recommendation of interest (top right) and view the patient-individual applicability and adherence of the recommendation on all current patients on a selected ward (left), where recommendation adherence is marked by check marks on green background. To allow the user to understand and review the results of the guideline recommendation evaluation, the user can select individual patients to show the original patient data required to assess the recommendation’s applicability and adherence (right). ARDS: acute respiratory distress syndrome; ICU: intensive care unit.

For our exemplary prototype, we designed and implemented a user interface aimed at assisting supervising medical staff in their task to review whether specific guideline recommendations are applicable and adhered to in individual patients who are treated in the wards for which they are responsible (Figure 4). The user interface designed for this specific task allows the user to select the guideline recommendation to check and then, along with an overview of the patients currently treated in the ward, gives a condensed evaluation to which of the patients this specific recommendation is applicable and in which of the patients it is adhered to. Furthermore, the user interface allows the user to view the patient data on which the guideline recommendation evaluation was performed to allow the clinician to review the evaluator’s results. The user interface is implemented as a dashboard website using RShiny [26], but it is easily exchangeable by any other user interface framework or implementation because of the REST API interface of the user interface backend through which it receives data.

#### Data Protection

The software was implemented as a containerized microservice architecture for effortless on-premises deployment within the IT infrastructure of individual hospitals. This ensures that the patient data do not leave the hospital’s network and that the data are therefore subject to the same regulation, such as the General Data Protection Regulation in the European Union, as the existing EHR system from which the patient data are used. As long as the CDS system developed here is used within or on top of existing EHR systems in the hospital, no further General Data Protection Regulation constraints other than those already governing the use of patient data for routine clinical practice are in place. As the system is intended to be run on-premises within the hospital’s IT network, the data are protected by the hospital’s existing security measures such as firewalls and intrusion detection systems. In our prototype implementation, the only interface to access data is the user interface backend, which provides user-level authentication to ensure that only authorized users can access the output of the system. Depending on the actual deployment scenario, each microservice can be augmented by an authentication scheme, and user access should be integrated with the organizational active directory or single sign-on system to authenticate users and grant access according to their roles and permissions within the organization.

### Demonstration of Prototype Utility

**Figure 5:**
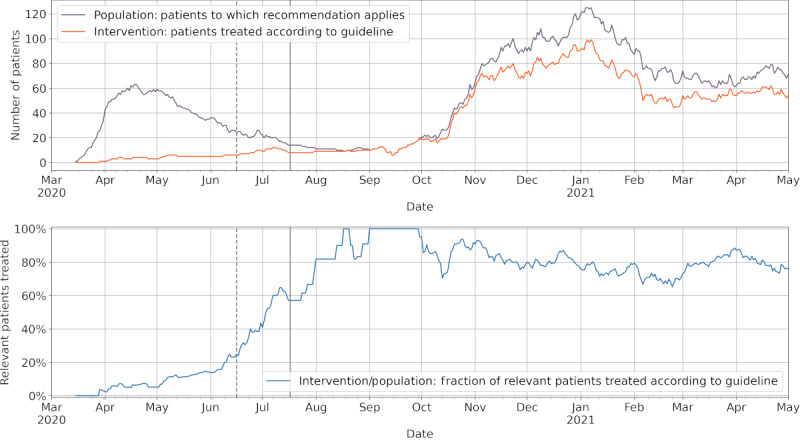
Individual applicability and adherence to a guideline recommendation for treating patients with severe or critical COVID-19 using steroids. Top: shown are the number of patients to which the guideline recommendation is applicable (purple) and which have been treated according to the guideline recommendation (orange) between March 2020 and May 2021. Bottom: shown are the number of patients that were treated according to the guideline recommendation as a fraction of patients to which the guideline recommendation is applicable. Vertical gray lines indicate date of the first press release of the detected dexamethasone efficacy in the RECOVERY trial (dashed line [47]) and the initial publication of the trial results (solid line [48]).

To demonstrate the utility of our prototype implementation, we have specified a recent evidence-based recommendation for the administration of dexamethasone to critically ill patients with COVID-19 from the guideline for inpatient treatment of patients with COVID-19 as a machine-readable guideline recommendation [44-46]. We connected the prototype to the critical care information systems and clinical information systems (CISs) of Charité Universitätsmedizin Berlin using a site-specific implementation of the clinical data interface. An exemplary time-dependent analysis of the applicability and adherence to guideline recommendations is shown in Figure 5 [47,48].

## Discussion

### Principal Findings

In this study, we demonstrated the system architecture and prototype implementation of a CDS system that automatically integrates clinical guideline recommendations with real-time clinical data to assist health care professionals by visualizing whether guideline recommendations apply to individual patients and whether the guidelines recommendations have been followed in individual patients. We described our stepwise approach for the development of the system, including the core requirements that shaped our software architectural design as well as our prototype implementation and demonstrated the prototype’s utility using a COVID-19 treatment guideline recommendation on clinical data.

To evaluate our architecture and prototype, we implemented a recent clinical guideline recommendation on the treatment of patients with severe or critical COVID-19 and integrated the recommendation with data from a large university hospital to analyze guideline recommendation adherence over time (Figure 5 [47,48]). The integration of the machine-readable guideline recommendation with clinical data could accurately detect the first and second waves of COVID-19 intensive care treatments [49] and the successful implementation of the guideline recommendation for the second wave, as seen by >70% of relevant patients treated according to the recommendation. The non-100% guideline recommendation implementation in our specific data set is primarily because a large proportion of patients with COVID-19 treated in this university medicine center were transferred from other hospitals and were treated with steroids in accordance with the guideline recommendations before arriving at the intensive care unit of our hospital [50]. Such a situation in which the recommended treatment has already taken place but was recorded in a different patient data management system could be solved by increased interoperable data exchange between different health care providers.

An automated integration of guideline recommendations with clinical data, as done by our developed systems, has several advantages: the system can provide a certain kind of decision support during individual patient treatment by pointing to applicable guideline recommendations, which the treating health care professionals might either not be aware of or which are known but whose applicability might go unnoticed. In addition, the monitoring of guideline recommendation adherence across groups of patients provides possibilities for their use as quality and performance indicators [51,52] that can easily be monitored in real time using a system, as we propose here. Another similar aspect could be the application of the system to monitor the process implementation of new guidelines and to provide clinical insights into the applicability of guideline recommendations that are useful for guideline updates. Independent of official clinical guidelines, the system can also easily be used to monitor hospital-specific treatment standards by formulating them as machine-readable guideline recommendations and providing them via the guideline interface to the adherence evaluator.

One of the key considerations in the design of our system is the separation of the contents of the clinical guidelines from the CDS software that integrates them with patient data. This approach has several advantages, including the separation of concerns; guideline developers can focus on the development of guidelines, whereas software developers can focus on the development of software. This allows for rapid updates of the guidelines, particularly for living guidelines that are continuously updated. However, this approach also has drawbacks. For example, rapidly changing guidelines may cause confusion for clinical staff if the system reports that patients are not being treated according to the current guideline simply because a new version of the guideline has been released and the treatment recommendation has changed. In addition, if the guidelines contain new decisions, items, or conditions that have not been covered by the execution engine code, the execution engine will not be able to process these updated guidelines.

We designed our system to require machine-readable guideline recommendations; however, guideline recommendations are available only in narrative, human-readable formats. Thus, individual guideline recommendations must first be converted into a machine-readable format which adds an extra amount of work. However, the specification of guideline recommendations in a machine-readable format enforces a precise and accurate formulation of guideline recommendations, which prevents ambiguities, as these cannot (easily) be resolved or understood by a software system. In addition, in contrast to converting human-readable recommendations into machine-readable recommendations, the generation of precise human-readable formulations of a guideline recommendation formulated in a machine-readable standard is a comparably simple task. Therefore, specifying guideline recommendations from the start in a machine-readable format has multiple advantages, and we consider it a desirable change in the current practice of high-quality evidence-based guideline recommendation development. An alternative approach to using a machine-readable guideline recommendation specification could be the application of recent advances in natural language processing methods to allow the computer to understand and process human-readable guideline recommendations [53,54]. However, any errors unknowingly introduced by such an approach (eg, because of imperfect understanding of the guidelines by natural language processing algorithms) could have severe consequences for patient health and outcomes. Therefore, we believe that the manual and explicit statement of guideline recommendations in a standardized machine-readable format, as used by our system, is currently the most suitable choice.

In our prototype implementation, we used an EBMonFHIR-based and CPG-on-FHIR-based representation of clinical guideline recommendations that covers the entire development process of evidence-based guidelines, from the underlying systematic review of available evidence on the rating of the individual evidence to the final recommendation in a computer-interpretable manner [23]. Computer-interpretable links to the development process are particularly useful in meeting the requirements of the system to adapt to updated guideline recommendations (requirement 3), as updates to recommendations based on new evidence or reappraisal of existing evidence can be automatically passed to the system without the need for new and manual conversion of human-readable guideline recommendations into representation formalism. Although requirement 3 could, in principle, be met by any guideline recommendation formalism that is semantically correct, complete, and unambiguous, the CPG-on-EBMonFHIR representation offers an advantage, especially with guideline recommendations that are updated regularly, such as recommendations from living guidelines. In addition, the CPG-on-EBMonFHIR representation could allow users of the software system to evaluate the certainty of the evidence and the evidence to decision process underlying individual recommendations. We did not include this functionality in the prototype implementation; however, it may become part of a later extension stage of the system implementation. Although a key idea behind the system is that guidelines can be updated efficiently and independently of human intervention, we have not performed a formal analysis of compute time dependencies for updates to the guidelines, as our focus was on developing the system requirements and demonstrating the technical feasibility of the system. However, if the guidelines are formulated in the FHIR-based standardized format, they can be updated, in principle, with time lags arising only from conceptually new recommendations or human processes and not from technical limitations.

This project aimed to create a system that specifically supports intensive care physicians’ work processes. However, we understand that different specialties or user groups may have unique approaches and techniques for managing various conditions and therefore may require different interfaces and work process integrations. To address this issue, the proposed system was designed with a modular architecture. This allows for the development of diverse user interfaces for various user groups, specialties, and disease models while maintaining the core functionality of checking guideline recommendations against patient data. This approach eliminates the need to build a new system for each application. Here, we developed a single prototypical user interface for demonstration purposes in the form of a dashboard website to demonstrate its feasibility. However, in clinical practice, it might be desirable to integrate the suggestions of guideline recommendations into the CIS implemented in the ward. For example, the system could be integrated into an EHR system user interface by highlighting laboratory values that fall outside of ranges supported by evidence-based recommendations and then providing the actual narrative recommendation and its underlying evidence on demand to the user. Owing to the separation of the user interface and backend in our system, these integrations can be readily implemented depending on the CIS, for example, as SMART-on-FHIR CDS hooks [55,56], if the CIS provides a SMART-on-FHIR interface.

In terms of the range of applicability of our system, we believe that it has the potential to be used in various medical specialties and clinical settings. The system is based on the use of evidence-based guideline recommendations, which are applicable across many different areas of medicine. Specifically, the system was designed to support the implementation of evidence-based guideline recommendations in critical care medicine, where the complexity and multiplicity of simultaneously applicable recommendations can make it challenging for health care professionals to correctly recognize every situation in which these recommendations should be applied. However, the system architecture is modular and independent of a particular user interface, which means that it can be adapted to support the implementation of guideline recommendations in other specialties and settings, such as primary care. Therefore, the range of applicability of the software can be broadened to other specialties and care settings. The main focus of the system is guideline-based CDS, and the actual implementation and integration of the system into specific clinical workflows are left to concrete implementation projects. In general, any guideline recommendation that can be checked using electronically recorded data can be used by a system such as the one demonstrated here.

We designed our software system to retrieve guideline recommendations from a centralized repository that could be hosted by medical societies or national standardization organizations. This allows societies or organizations to develop guideline recommendations independently of our system, thereby focusing on their expertise. Once new or updated guideline recommendations are published by medical societies or organizations on their servers, they can be automatically retrieved using our system and integrated with clinical data. However, a checkpoint in this process should be established, where health care professionals of individual hospitals first review new or updated guideline recommendations retrieved from the central repository before releasing them for implementation in their hospital. This helps mitigate risks that arise if the central guideline recommendation server is compromised by malicious attackers and ensures that the guideline recommendations implemented in the hospitals are in accordance with the hospitals’ policies.

The primary goal of the study was to derive the requirements for a system for automated guideline-based CDS and to develop a prototype and evaluate its technical feasibility. Although we have not yet conducted a formal evaluation of the system’s impact on clinical practice and patient outcomes, we believe that the system has the potential to improve patient care by assisting health care professionals in making informed decisions based on the latest evidence-based guideline recommendations. Studies have consistently shown that the implementation of guideline-based CDS systems can potentially improve the quality of care [57], increase guideline adherence [58,59], and positively impact the care process [60,61]. However, it should be noted that larger trials are needed to confirm these findings and that it is crucial to improve the interoperability of CISs to establish a widespread use of CDS systems, such as those developed by us [59]. The system developed in this study could help overcome some of the challenges commonly encountered in the implementation of guideline-based CDS systems. Specifically, the system’s ability to automatically process updated machine-readable guidelines as they are published in appropriate repositories and integrate them with standardized patient data could improve the timeliness and completeness of decision support, which, in turn, could lead to more accurate and consistent patient care and potentially better patient outcomes. However, further research is needed to confirm this potential impact and assess the system’s effectiveness of the system in different clinical settings.

### Comparison With Prior Work

Developing a system similar to ours, which integrates guideline recommendations with real-time clinical data, is a complex task that requires expertise from a variety of different fields. In addition to guideline experts, it also requires input from clinical experts, as well as specialized expertise in areas such as implementation, clinical data management, and IT infrastructure. The COVID-19 pandemic provided a unique opportunity for us to develop such a system and enabled us to bring together a wide range of expertise within the framework of a well-funded federal project, including the EBM-on-FHIR initiative, the German Association of the Scientific Medical Societies, which maintains the central German guideline register, and Cochrane Germany, to successfully develop and implement our system, thanks to the collaborative efforts of all stakeholders involved. Other initiatives aimed at improving the implementation of evidence-based guidelines in clinical practice include the CDS Connect project, led by the Agency for Healthcare Research and Quality of the US Department of Health [62], which focuses on developing and promoting the use of standards-based, computable clinical guidelines and knowledge artifacts in CDS systems. Another initiative is the Guideline Definition Language of the openEHR foundation, which allows expressing the decision logic for integration with the openEHR reference model [63]. These initiatives have similar goals to our system but focus on the development and sharing of computable knowledge artifacts, whereas our system focuses on the automated integration of evidence-based guidelines with patient data. Another initiative, on which we have built on, is the CPG-on-FHIR project, which provides an extensive methodology on how to represent guidelines in a computer-interpretable fashion [64], leveraging the Clinical Quality Language for creating reusable computable knowledge artifacts; however, to our knowledge, no system has been developed to integrate these guidelines with patient data. However, an execution engine for Clinical Quality Language artifacts is currently being developed [65].

### Limitations

The study and CDS system developed here have certain limitations that should be considered when evaluating its usefulness and applicability in clinical practice.

First, the system is currently focused on evidence-based guideline recommendations, which are simple rules linking specific conditions to specific actions, as they are typically derived from clinical studies with specific interventions and a limited number of options. Complex multistep clinical paths, consisting of decision points and possible actions that require user feedback, are not currently supported by the system. However, the CPG-on-FHIR standard on which we built the CPG-on-EBMonFHIR representation can represent complex multistep guidelines, and our system may be developed to support these more complex clinical workflows in the future [23,64].

Second, the system was designed to work with evidence-based guideline recommendations that are represented in a machine-readable format. However, evidence-based guideline recommendations have been developed for human-readable narrative statements. Representing them in a machine-readable format, such as EBMonFHIR or CPG-on-FHIR, poses a series of challenges and requires a significant amount of manual work and expertise from several groups of experts, such as guideline developers, knowledge engineers, and clinical experts [66]. In addition, not all guideline recommendations are suitable for representation in this format, which may limit the applicability of the system in certain situations. However, we believe that with ongoing international efforts to standardize health care data and interoperability with projects such as EBMonFHIR and the increased usability of artificial intelligence–based support tools for knowledge acquisition and representation, computable evidence will be available at scale in the future, which will minimize the need for manual translation of narrative guidelines into computer-interpretable representations.

Third, the system can only handle recommendations that can be evaluated using data from the hospital’s EHR system, which may limit its applicability in situations where other types of data or information are needed to make clinical decisions (eg, clinician instrumentation, patient preferences, or infrastructural settings).

Fourth, the system cannot consider uncertainties in the recommendations, which may impact its ability to provide appropriate guidance in certain cases. Furthermore, if the data used by the system are incorrect, this may lead to false alarms, which could negatively affect the trust of clinical staff in the system.

Fifth, the system currently cannot ask for feedback or input from human users. It is fully automated, which may lead to a lack of flexibility in certain cases.

In conclusion, although the CDS system described in this paper has the potential to improve adherence to guideline recommendations in clinical practice, the limitations described earlier should be considered when evaluating its usefulness and applicability in real-world settings.

### Conclusions

In this study, we designed a system that integrates guideline recommendations with real-time clinical data to evaluate adherence to individual guidelines and to develop a functional prototype. The proposed system has advantages for both the individual treatment of patients, as clinical guidelines condense the current state of medical knowledge into treatment recommendations and for the quality management and monitoring of patient treatment standards in hospitals, which has the potential to improve the implementation of evidence-based guidelines and ultimately improve patient outcomes. However, further studies are required to quantify the specific impact of the system on patient outcomes. In addition, further research is needed to evaluate the resource effectiveness of the system and its potential for implementation in different clinical settings and specialties. We have specified a modular software architecture where each module corresponds to a particular area of expertise, allowing experts from different fields (guideline developers, software engineers, medical data engineers, and health care professionals) to work independently and focus on their area of expertise. We have released the source code of our system under an open-source license (see Data Availability) and invited for cooperation and collaborative further development of the system [67].

## Appendix

Multimedia Appendix 1 Technical details of the prototype implementation.

## Abbreviations

API: application programming interface
CDS: clinical decision support
CIS: clinical information system
CPG-on-EBMonFHIR: Clinical Practice Guidelines on Evidence-Based Medicine on Fast Healthcare Interoperability Resources
EHR: electronic health record
FHIR: Fast Healthcare Interoperability Resources
PICO: population or patients, intervention, comparison, outcomes

## Data Availability

The complete code for the clinical guideline recommendation system was published under an open-source license (AGPL version 3.0) [68]. Application programming interface–level documentation can also be found [69].
